# Temporal Trends and Demographic Contributions to Stroke Burden in India, 1990–2023: An Analysis of Global Burden of Disease 2023 Estimates Using Shapley Decomposition and Joinpoint Regression

**DOI:** 10.7759/cureus.117056

**Published:** 2026-09-28

**Authors:** Rajan Kaushik, Soorveer Singh Gurjar, Athira K R

**Affiliations:** 1 Community Medicine, Adesh Medical College & Hospital, Mohri, IND; 2 Community Medicine, Autonomous State Medical College and Hospital, Lalitpur, IND; 3 Community Medicine, Amala Institute of Medical Sciences, Thrissur, IND

**Keywords:** daly, decomposition analysis, gbd 2023, india, joinpoint analysis, stroke, temporal trends

## Abstract

Background: Stroke is an important cause of death and disability in India, with demographic change potentially obscuring improvements in age-standardized outcomes.

Objectives: The objectives of this study were to assess temporal trends in stroke incidence, prevalence, mortality, and disability-adjusted life years (DALYs) in India from 1990 to 2023; to describe age-, sex-, geographic-, and stroke subtype-specific patterns; and to quantify the contributions of population growth, population ageing, and epidemiological change to changes in the absolute stroke burden.

Methods: Global burden of disease (GBD) 2023 estimates for India were analysed for 1990-2023. Absolute number of cases and crude and age-standardized rates were examined by age, sex and geographic location. Joinpoint regression assessed temporal trends in age-standardized incidence, mortality, and DALY rates, while Shapley decomposition quantified the contributions of growing population, population ageing, and epidemiological change.

Results: Incident stroke cases rose from approximately 0.73 million to 1.87 million (an increase of 155.1%), deaths from approximately 0.42 million to 1.03 million (an increase of 148.2%), and DALYs from 12.41 million to 25.99 million (109.4%). Despite this increase, age-standardized incidence remained stable (average annual percent change (AAPC), −0.0195%; P=0.079584), whereas age-standardized deaths (AAPC, −0.4013) and DALY rates (AAPC, −0.5393) declined significantly (both P<0.001). Burden of stroke increased with age and was generally higher among males, with substantial geographic heterogeneity. A growing and ageing population was the major contributor to the rise in incidence, deaths, and DALYs, while epidemiological change partially offset these effects.

Conclusion: India's stroke burden is shaped by population growth and ageing rather than worsening age-standardized risk alone. Declines in age-standardized mortality and DALY rates represent favourable changes in these measures of stroke burden, while the increasing prevalence indicates that improvements have not been uniform across all epidemiological measures. The growing absolute burden underscores the need for intensified risk-factor prevention alongside continued expansion of acute stroke care, rehabilitation, and long-term support.

## Introduction

Stroke is a major neurological and cardiovascular disease and also one of the leading causes of mortality and long-term disability globally. It is defined as a sudden neurological deficit caused by disrupted blood flow to the brain and is mainly divided into ischemic stroke and hemorrhagic stroke. Ischemic stroke occurs when a cerebral artery is blocked and accounts for the largest number of new stroke cases worldwide. Hemorrhagic stroke happens when a blood vessel in the brain bursts and includes intracerebral hemorrhage (ICH) and subarachnoid hemorrhage (SAH). According to estimates from the Global Burden of Disease (GBD) study 2019, ischemic stroke accounts for about 62.4% of all new strokes, compared to 27.9% for ICH and 9.7% for SAH [1]. Although ischemic stroke is the most common type by incidence, the hemorrhagic subtypes cause a disproportionate amount of premature death and disability, highlighting the need to view stroke as a diverse group of diseases rather than a single clinical condition [1,2].

From an epidemiological standpoint, the importance of stroke extends beyond mortality because it often leads to long-term neurological deficits and losses in functional capacity. To understand the disease's impact fully, it is necessary to consider various facets of disease burden, including incidence, prevalence, mortality, years of life lost (YLLs), years lived with disability (YLDs), and disability adjusted life years (DALYs). Since DALYs account for both premature death and disability, they provide a more complete measure of disease burden than mortality alone. These measures can show opposite trends, as improvements in survival may cause mortality to fall while the number of people living with stroke-related disability increases. An analysis of ischemic stroke based on GBD 2019 data in India showed that new cases, deaths, YLLs, and DALYs all declined between 1990 and 2019, even though DALY and YLD rates increased [3]. This difference highlights the importance of examining both absolute burden and standardized rates when assessing changes in stroke burden.

Stroke imposes substantial health and socioeconomic consequences, and understanding how stroke burden changes over time is particularly important in countries undergoing rapid demographic and epidemiological transitions. India represents an important setting for examining these patterns because of its large and ageing population, substantial regional and socioeconomic heterogeneity, and the coexistence of established and emerging cardiovascular risk factors. Stroke makes up a large part of the country’s neurological disease burden and shows differences across regions. The India State-Level Disease Initiative reported that stroke made up 37.9% of neurological-disorder DALYs in 2019, and crude stroke DALY rates varied by about 5.5 times between states [4]. Earlier state-level studies of cardiovascular diseases found about six-fold differences in stroke DALY rates across Indian states [5]. These differences show that a single national estimate cannot capture the full disease burden. Instead, factors like demographics, socioeconomic status, environment, behaviour, and the health system may all affect how stroke is distributed and progresses in India.

Stroke burden tracking over time in India is complex because changes in stroke numbers do not always match changes in risk. Even if age-specific or age-standardized rates (ASRs) go down, the total number of stroke cases can still rise due to ageing and population growth. Global GBD studies show that even when ASRs drop, a large population and an older population can still cause absolute DALYs and deaths to increase [6]. India shows a similar pattern. A GBD 2019 study found that the ASR in stroke incidence dropped from 121.35 per 100,000 in 1990 to 110.7 per 100,000 in 2019, an average yearly decrease of 0.32% [7]. Still, the total number of stroke cases went up during this time. It is important to consider both demographic and epidemiological changes, not just crude counts or standardized rates, to fully understand India’s stroke epidemic.

The GBD framework is a useful tool for measuring disease burden because it uses a standard modelling method to estimate this burden across different countries, states, age groups, sexes, and time periods. GBD 2023 provides estimates for many diseases and injuries, drawing on data from sources like vital registration, surveillance systems, surveys, censuses, verbal autopsies, and other available information. GBD 2023 uses the Cause of Death Ensemble model (CODEm) and other statistical methods, taking into account differences in data quality and availability to estimate cause-specific mortality. The 2023 update also added new data and made methodological changes, including adjustments for misclassified COVID-19 deaths, which led to a re-estimation of past data. GBD 2023 now serves as an updated reference for studying long-term stroke trends up to 2023. When comparing it to earlier GBD versions, differences may show real changes over time or updates to the models used [8].

Age acts as a key demographic factor in stroke risk. Both the number of cases and death rates from stroke increase as people age, although exact patterns can vary. The GBD study found that older adults have much higher stroke mortality than younger people [9]. However, stroke in younger adults need special attention because it causes long-term disability and loss of productivity during working years of life. International GBD data show that ischemic stroke in younger adults may have different risk patterns than in older groups [10]. These results support using age-specific assessments instead of assuming stroke risk factors are the same at all ages. Sex is another important factor in stroke risk. Data from India show that men currently have higher rates of stroke, death, and DALYs, but trends over time can differ between men and women [9]. The GBD 2019 analysis found that the increase in stroke cases and deaths was greater among women, even though men still had higher overall rates [3]. Differences in baseline risk, age, exposure, and survival may explain these patterns. It is important to examine both absolute and relative changes by sex to identify which groups are seeing the rapid rise in stroke burden.

Previous research has improved understanding of stroke in India. The GBD 2016 study found significant differences in cardiovascular risk factors across states and tracked changes over time [5]. Another study reviewed neurological disorders in different states and confirmed stroke as the main cause of neurological DALYs [4]. Another study used GBD 2019 data to study stroke incidence with age-period-cohort and joinpoint methods and found a drop in age-standardized incidence and identified periods when trends shifted [7]. A more recent analysis of GBD 2021 data examined patterns by age, sex, and risk factors, and highlighted that while the overall burden is rising, rates are falling in some age groups [9]. The National Stroke Registry Programme provides population-based data from five regions, showing ischemic stroke is most common and that incidence and case fatality differ between rural and urban areas [11]. Together, these studies reveal that stroke in India varies widely by region and demographic group and that the relationship between total burden, standardized rates, and risk factors is complex.

Research gaps are still present in the current understanding of stroke epidemiology in India. Much of the existing research relies on earlier versions of the GBD study, primarily 2016, 2019, and 2021. The most recent GBD 2023 release incorporates updated data and methodologies, offering a more robust foundation for analyzing trends from 1990 to 2023 [8]. Previous studies have typically examined descriptive trends, demographic breakdowns, or risk factors in isolation, rather than integrating these elements to assess the contributors to changes in the overall stroke burden. Demographic change is recognized as a major factor in the increasing number of stroke cases [12]. However, relatively few studies have explicitly quantified the relative contributions of population growth, population ageing, and epidemiological change to the increase in absolute stroke burden. Prior joinpoint analyses have identified inflection points in stroke rate trends, but they have not consistently linked these findings to a decomposition of changes in the total burden. Joinpoint regression is used to detect shifts in trends, whereas decomposition analysis clarifies the demographic contributions and disease-related factors to overall change. These methods are complementary and should be applied in conjunction [7,12].

This study used the GBD 2023 estimates to examine trends and demographic factors influencing the stroke burden in India from 1990 to 2023. To analyse changes in the absolute burden, a temporal trend analysis was conducted. Joinpoint regression was conducted to identify significant changes in trend and to calculate annual percentage changes across different periods. Shapley decomposition was applied to attribute changes in stroke burden to demographic and epidemiological contributions, thereby facilitating an understanding of developments over the past three decades.

## Materials and methods

Study design

This was a secondary data-based, descriptive ecological study to assess the burden of stroke at national and subnational (state) levels in India from 1990 to 2023, utilizing estimates from the GBD 2023 study. Temporal trends in stroke incidence, prevalence, death, and DALYs were analysed. The analysis included variations by age and sex, geographic heterogeneity among GBD-defined Indian locations, burden of major stroke subtypes, and evaluation of demographic and epidemiological contributions to changes in stroke burden. The study used the Guidelines for Accurate and Transparent Health Estimates Reporting (GATHER) checklist for reporting study findings [13].

Data source

Data for this study were obtained from the GBD 2023 study via the Global Health Data Exchange (GHDx) [14]. The analysis focused on the combined "Stroke" cause category within the GBD cause hierarchy. Estimates for incidence, prevalence, death, and DALYs were extracted using the GBD Results Tool. Comma-separated value (CSV) datasets included variables such as location, year, cause, measure, metric, age group, sex, point estimate, and corresponding lower and upper uncertainty intervals (UIs). For national and subnational trend analyses of 31 GBD-defined Indian locations, ASRs with 95% UIs were obtained. Sex-specific estimates were calculated for males and females, while age-specific estimates corresponded to selected GBD-defined age groups. Population data were obtained from the GBD 2023 estimates for India, including age-specific population counts by selected age groups for 1990-2023; these data were combined with age-specific GBD stroke incidence, death, and DALY rates to decompose changes in stroke burden into factors from population growth, ageing, and epidemiological change. Data extraction was conducted on August 8, 2026. The specific extraction parameters are detailed in the Appendices to ensure reproducibility (Appendix A and B). Datasets were organized to facilitate national-level, age, sex, geographic location, and cause-specific analyses. Data processing and quality-control procedures were completed prior to statistical analysis. GBD estimates are periodically updated using revised data sources and modelling methods, so comparisons of estimates across different GBD rounds should be interpreted cautiously, as observed differences may partly reflect methodological revisions and re-estimation rather than true changes in the underlying burden.

Study outcomes

The stroke burden was assessed using four parameters: incidence, prevalence, deaths, and DALYs. For ischemic stroke, ICH, and SAH, we analysed each subtype using ASRs to highlight differences in epidemiological patterns between ischemic and hemorrhagic strokes. We examined both numbers and rates, with rates reported per 100,000 people. Crude rates showed the overall population burden, while ASRs allowed comparison over time and between populations having different age structures. All ASRs were obtained from the GBD database [14].

Geographic and temporal scope

Data were collected for India and the 31 GBD-defined Indian locations representing Indian states and union territories (UTs). The boundary dataset was obtained from open-access boundary shapefiles provided by the Survey of India. The GBD-defined combined Jammu & Kashmir and Ladakh location and Other UTs category were handled according to the geographic definitions used in the GBD 2023 database. Locations for which a directly corresponding geographic polygon was unavailable or where boundaries were disputed were excluded from the relevant maps rather than assigning estimates to an inappropriate polygon. India was the main focus, but we also examined data for each state and UT listed in the GBD 2023 database. The study covered 1990 to 2023, providing 34 years of data. We chose this period because comparable GBD data were available for all these years.

Data management

Data were imported from the GBD Results Tool into R version 4.6.1 (R Foundation for Statistical Computing, Vienna, Austria) for preprocessing and statistical analysis. We used the R packages 'dplyr', 'purrr', and 'sf' for data processing and filtering, and 'ggplot2', 'ggsci', 'ggrepel', 'patchwork', and 'ggbrace' for visualization. The data were filtered to include India, focusing on numbers, crude rates, and ASRs, including all available states and UTs from the GBD, with a main focus on ASRs. Separate datasets were prepared for national trends, state comparisons, sex, age, and Joinpoint regression. Joinpoint regression analysis was performed using the Joinpoint Regression Program, version 6.1.0 (released July 2026; Surveillance Research Program, National Cancer Institute (NCI), Bethesda, Maryland, United States). Before analysis, we checked all datasets for duplicates, missing data, coding errors, and completeness to ensure data quality and reproducibility.

Statistical analysis

Descriptive and Temporal Trend Analysis

The national stroke burden was described for 1990 and 2023 using absolute numbers, crude rates, and ASRs. Four outcomes were analysed: incidence, prevalence, deaths, and DALYs. Rates were expressed per 100,000 population. ASRs were used for temporal and geographical comparisons to minimize the influence of different population age structures. The percentage change between 1990 and 2023 was calculated as:

$\text{Percentage Change} = \frac{\mathrm{Estimate}_{2023} - \mathrm{Estimate}_{1990}}{\mathrm{Estimate}_{1990}} \times 100$

Annual time-series graphs were created using the age-standardized incidence, prevalence, death, and DALY rates for India from 1990 to 2023. We represented the uncertainty around each GBD estimate with its reported 95% UI. These intervals were treated as uncertainty intervals from the GBD modelling framework rather than conventional survey-based confidence intervals (CI). For Joinpoint regression analyses requiring a standard error (SE), assuming a symmetric normal approximation, the SE for each annual estimate was calculated as:

$\text{Standard Error} = \frac{\text{Upper UI} - \text{Lower UI}}{2 \times 1.96}$

The annual GBD point estimates and these approximated SEs were supplied to the Joinpoint Regression Program. This approach was adopted because Joinpoint requires an estimate of the uncertainty associated with each observation when using the SE-based heteroscedastic error option. The resulting SEs should be regarded as approximations of the uncertainty surrounding the GBD model estimates and not as conventional sampling SEs. Because covariance information between annual GBD estimates was not available in the extracted GBD data, potential serial dependence arising from the underlying modelling process could not be explicitly incorporated into the analysis. Therefore, annual percent change (APC) and AAPC estimates and their CIs should be interpreted in the context of this uncertainty approximation.

We examined sex-specific trends separately for males and females. Annual estimates from 1990 to 2023 were extracted for incidence, prevalence, deaths, and DALYs. Temporal comparisons between male and female populations were based primarily on ASRs, while point estimates and their 95% UIs were retained. Age-specific stroke burden was assessed for India using the selected GBD age categories available in the dataset: <5, 5-9, 10-14, 15-19, 20-24, 25-29, 30-34, 35-39, 40-44, 45-49, 50-54, 55-59, 60-64, 65-69, 70-74, 75-79, 80-84, 85-89, 90-94, and ≥95 years. ASRs were extracted separately for male sex, female sex, and both sexes. The age groups were ordered chronologically for graphical presentation.

For each state and UT available in GBD data, both-sex, age-standardized incidence, prevalence, death, and DALY rates were extracted for 1990 and 2023. States and UTs were ranked according to their ASRs to describe geographical heterogeneity. Geographical variation was visualized using choropleth maps (the type of map in which different geographic areas are shaded or colored according to the value of a particular variable). GBD location names were standardized before epidemiological estimates were merged with the geographical boundary files. Disputed geographical polygons (Combined Other UTs category) were excluded. Jammu & Kashmir and Ladakh were treated as a combined GBD analytical unit because the GBD dataset provides a combined estimate for these locations. Because the GBD database reports Jammu & Kashmir and Ladakh as a combined analytical unit, the combined estimate was not interpreted as a Ladakh-specific estimate. For cartographic display only, the combined value was mapped to the corresponding representative polygon.

Estimated Annual Percentage Change (EAPC) Analysis

Long-term trends in state- and UT-specific stroke burden were summarized using EAPC for incidence, prevalence, deaths, and DALYs over 1990-2023 by a log-linear regression model using time in calendar year as the independent variable and the natural logarithm of the ASR as the dependent variable [15].

$ln(ASR)=\alpha+\beta\times(YEAR)+\epsilon$

where α denotes the intercept, β represents the annual change (slope) in the natural logarithm of the ASR, and ε is the random error term. The EAPC was calculated as:

$EAPC=100\times (e^{\beta}-1)$

EAPC estimates were accompanied by 95% CIs and presented using forest plots, with zero representing no annual change. States and UTs were classified as having an increasing trend when the lower limit of the 95% CI was greater than zero, a decreasing trend when the upper limit was less than zero, and a stable trend when the 95% CI included zero.

Stroke Subtype Analysis

Stroke burden was examined separately for the three major stroke subtypes: ischemic stroke, ICH, and SAH. Subtype-specific estimates were extracted for India, both sexes combined, and ASRs from 1990 to 2023. Incidence, prevalence, death, and DALYs were assessed for each subtype, with corresponding 95% UIs.

Joinpoint Regression Analysis

Joinpoint regression analysis was used to identify changes in the temporal trajectory of national age-standardized stroke rates during 1990-2023. Separate analyses were conducted for incidence, deaths, and DALYs. The annual input dataset contained year, ASR, and derived SE. The derived SEs were used as approximations of the uncertainty of the annual GBD estimates for Joinpoint regression. The logarithm of the annual ASR was modelled as a function of calendar year using connected linear segments. For each segment, the APC was estimated, representing the average annual relative change in the rate during that segment. The AAPC was used to summarize the overall temporal trend across the complete study period. A positive APC/AAPC indicated an increasing trend, whereas a negative value indicated a decreasing trend. A trend was classified as statistically significant when its 95% CI did not include zero. Trends for which the 95% CI included zero were classified as stable. Joinpoints were interpreted as changes in the statistical trajectory of the time series. The Joinpoint regression model specifications and statistical settings used for the temporal trend analyses are presented in the Appendices (Appendix C).

Shapley Decomposition Analysis

To measure the relative contribution of demographic and epidemiological factors to the observed change in the absolute stroke burden between 1990 and 2023, a Shapley decomposition analysis was conducted. The analysis was performed separately for stroke incidence, stroke deaths, and stroke DALYs. The decomposition was designed to differentiate the effects of three components: population growth, population ageing, and epidemiological change. For each year (t) and age group (a), three quantities were defined: total population (P_t_), the proportion of the population belonging to age group (a), denoted by (S_a_), and the corresponding age-specific disease rate (R_a_). Total population was calculated as:

$P_{t}=\sum_{a}P_{a,t}$

where: (P_t_) = total population in year (t) and (P_a,t_) = population in age group (a) in year t.

The proportion of the population in each age group was then calculated as:

$S_{a,t}=\frac{P_{a,t}}{P_{t}}$

Thus, (S_a,t_) represents what fraction of the total population belongs to age group (a). The expected absolute number of stroke events was calculated by combining population size, age structure, and age-specific stroke rates:

$D(P,S,R)=\sum_{a}\frac{P\times S_{a}\times R_{a}}{100000}$

Where (D(P,S,R)) = expected absolute stroke burden, (P) = total population, (S_a_) = proportion of the population in age group (a), (R_a_) = stroke rate in age group (a), and 100,000 was used as the denominator because the rates were expressed per 100,000 population. The same equation was used for incidence, deaths, and DALYs, with the corresponding age-specific rate substituted for (R_a_).

The total change in absolute stroke burden was calculated as:

$\bigtriangleup D=D_{2023}-D_{1990}$

where: (D_1990_) = estimated absolute stroke burden in 1990, (D_2023_) = estimated absolute stroke burden in 2023 and (ΔD) = total change in burden between 1990 and 2023. The decomposition considered three components: population growth, defined as the effect of change in total population size; population ageing, defined as the effect of change in the population age structure; and epidemiological change, defined as the effect of changes in age-specific stroke rates [16]. Because the contribution of a component can depend on the order in which components are introduced, all six possible permutations of the three components were evaluated. These were population growth-ageing-epidemiological change, population growth-epidemiological change-ageing, ageing-population growth-epidemiological change, ageing-epidemiological change-population growth, epidemiological change-population growth-ageing, and epidemiological change-ageing-population growth. For each permutation, the marginal contribution of each component was calculated as the difference in reconstructed burden before and after changing that component, while the other components were held constant. The final Shapley contribution for each component was then calculated as the arithmetic mean of its marginal contributions across the six permutations:

$C_{j}=\frac{1}{6}\sum_{k=1}^{6} MC_{jk}$

where (C_j_) represents the Shapley contribution of component (j), and (MC_jk_) represents its marginal contribution in permutation (k). The percentage contribution of each component to the overall change in burden was calculated as:

$Contribution (Percentage)=\frac{C_{j}}{\bigtriangleup D}\times 100$

Thus, the relative contribution of population growth, ageing, and epidemiological change could be expressed as percentages of the total change in absolute stroke burden. The decomposition was checked for internal consistency by verifying that the sum of the three Shapley contributions reproduced the observed change:

$\bigtriangleup D=D_{growth}+D_{ageing}+D_{epidemiological}$

The Shapley decomposition was interpreted as an accounting decomposition rather than a causal analysis. Accordingly, the population-growth component represents the contribution of changes in population size, the ageing component shows the contribution of changes in age composition, and the epidemiological component shows the contribution of changes in age-specific stroke rates [17]. The same procedure was then applied independently to incidence, deaths, and DALYs using their respective age-specific rates.

Ethical considerations

This study utilized publicly available, aggregated, and modelled GBD estimates. The study involved no direct interaction with human participants and no access to identifiable individual-level data. Therefore, approval from an Institutional Ethics Committee and informed consent were not required, following both institutional and national guidelines.

## Results

Temporal trends in stroke burden

From 1990 to 2023, the total burden of stroke in India has increased. The number of new cases went up from 732,142 in 1990 to 1.87 million in 2023 (an increase of 155.1%), and the number of existing cases rose from 5.47 million to 13.64 million (an increase of 149.2%). Stroke deaths also increased during this time, rising from 416,863 to 1.03 million (a rise of 148.2%), and DALYs moved from 12.41 million to 25.99 million (an increase of 109.4%) (Table 1).

**Table 1:** Stroke burden in India in 1990 and 2023: absolute numbers, crude rates, age-standardized rates and percentage change Crude rates and ASRs are expressed per 100,000 population. Estimates were obtained from the Global Burden of Disease (GBD) Study 2023 [14]. ASR: age-standardized rate; DALYs: disability adjusted life years; UI: uncertainty interval

Measure	Metric	Year 1990, point estimate (95% UI)	Year 2023, point estimate (95% UI)	Percentage change
Incidence	Number	732,142 (645,793–824,341)	1,867,893 (1,673,253–2,101,019)	155.1
	Crude rate (per 100,000)	86.61 (76.39–97.51)	129.41 (115.93–145.56)	49.4
	ASR (per 100,000)	151.73 (133.57–172.39)	151.66 (135.96–170.55)	-0.1
Prevalence	Number	5,473,310 (4,924,370–6,054,241)	13,642,041 (12,375,749–15,025,015)	149.2
	Crude rate (per 100,000)	647.46 (582.52–716.18)	945.14 (857.41–1,040.96)	46.0
	ASR (per 100,000)	952.56 (848.92–1,054.39)	1,014.38 (918.53–1,119.08)	6.5
Deaths	Number	416,863 (320,764–534,064)	1,034,796 (830,428–1,261,720)	148.2
	Crude rate (per 100,000)	49.31 (37.94–63.18)	71.69 (57.53–87.41)	45.4
	ASR (per 100,000)	104.74 (80.44–134.49)	92.88 (74.42–112.97)	-11.3
DALYs	Number	12,413,720 (9,740,388–15,805,038)	25,989,388 (20,479,957–31,670,577)	109.4
	Crude rate (per 100,000)	1,468.46 (1,152.22–1,869.63)	1,800.59 (1,418.88–2,194.19)	22.6
	ASR (per 100,000)	2,500.83 (1,956.71–3,172.67)	2,085.33 (1,650.56–2,520.90)	-16.6

Even though the total burden had increased, the age-standardized trends were very different. The age-standardized incidence rate was almost the same in the period from 1990 to 2023 (151.73 as compared with 151.66 per 100,000; a decrease of −0.1%), while the age-standardized prevalence rate rose by 6.5%. On the other hand, both the age-standardized death and DALY rates fell by 11.3% and 16.6%, respectively (Table 1, Figure 1). The incidence rate first decreased before increasing in the later years, whereas the death and DALY rates showed an overall downward trend (Figure 1).

**Figure 1:**
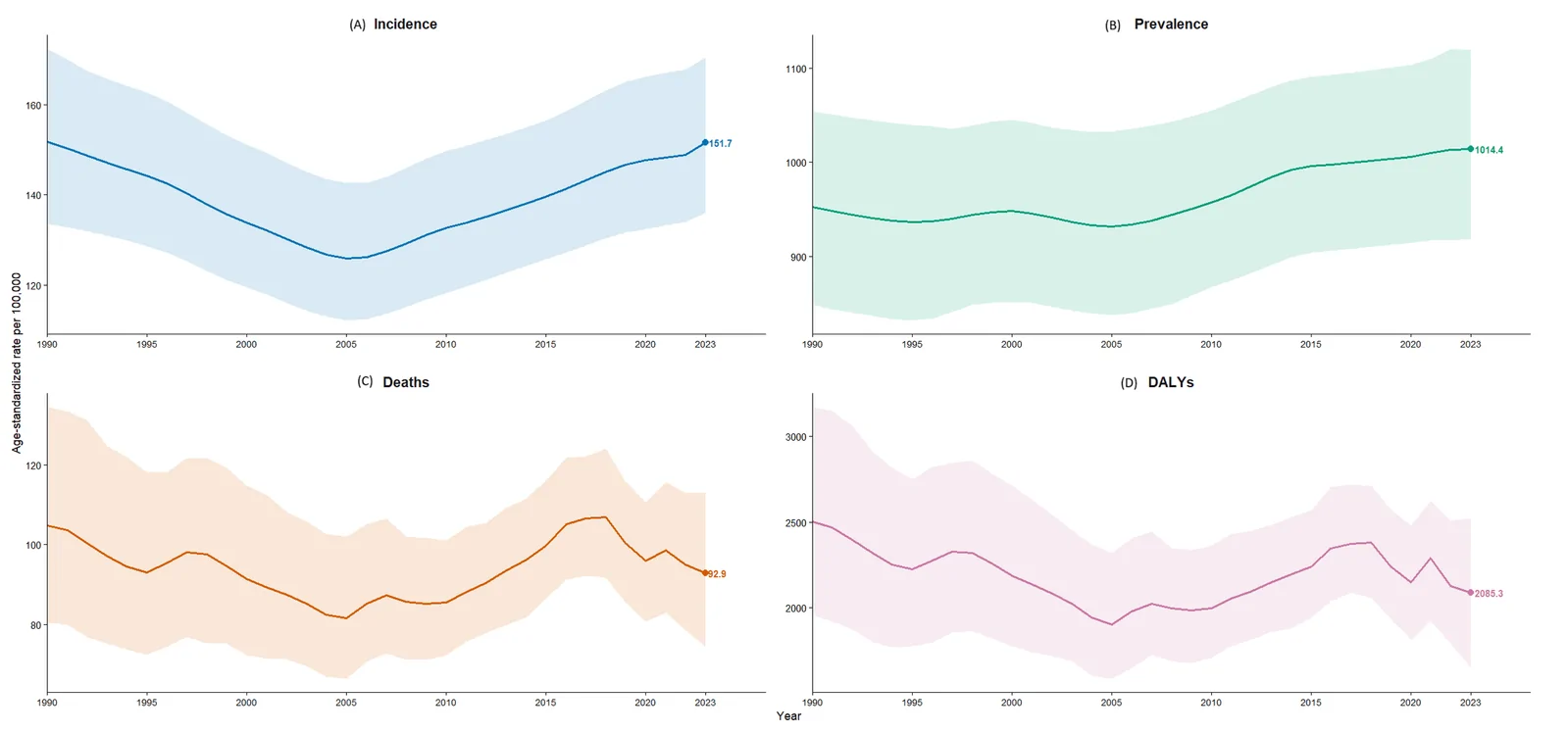
Temporal trends in age-standardized stroke (A) Incidence, (B) Prevalence, (C) Deaths and (D) DALYs rates, India, 1990–2023. Age-standardized rates are expressed per 100,000 population. Estimates were obtained from the Global Burden of Disease (GBD) Study 2023 [14]. Solid lines represent point estimates and shaded bands indicate 95% uncertainty intervals. DALYs: disability adjusted life years

Sex- and age-specific patterns

The burden of stroke was higher in males than in females, with the magnitude of the sex differential varying by outcome (Figure 2). While the extent of the difference varied, the temporal trends for both sexes were generally similar, showing an initial decrease in incidence followed by an increase in the later period.

**Figure 2:**
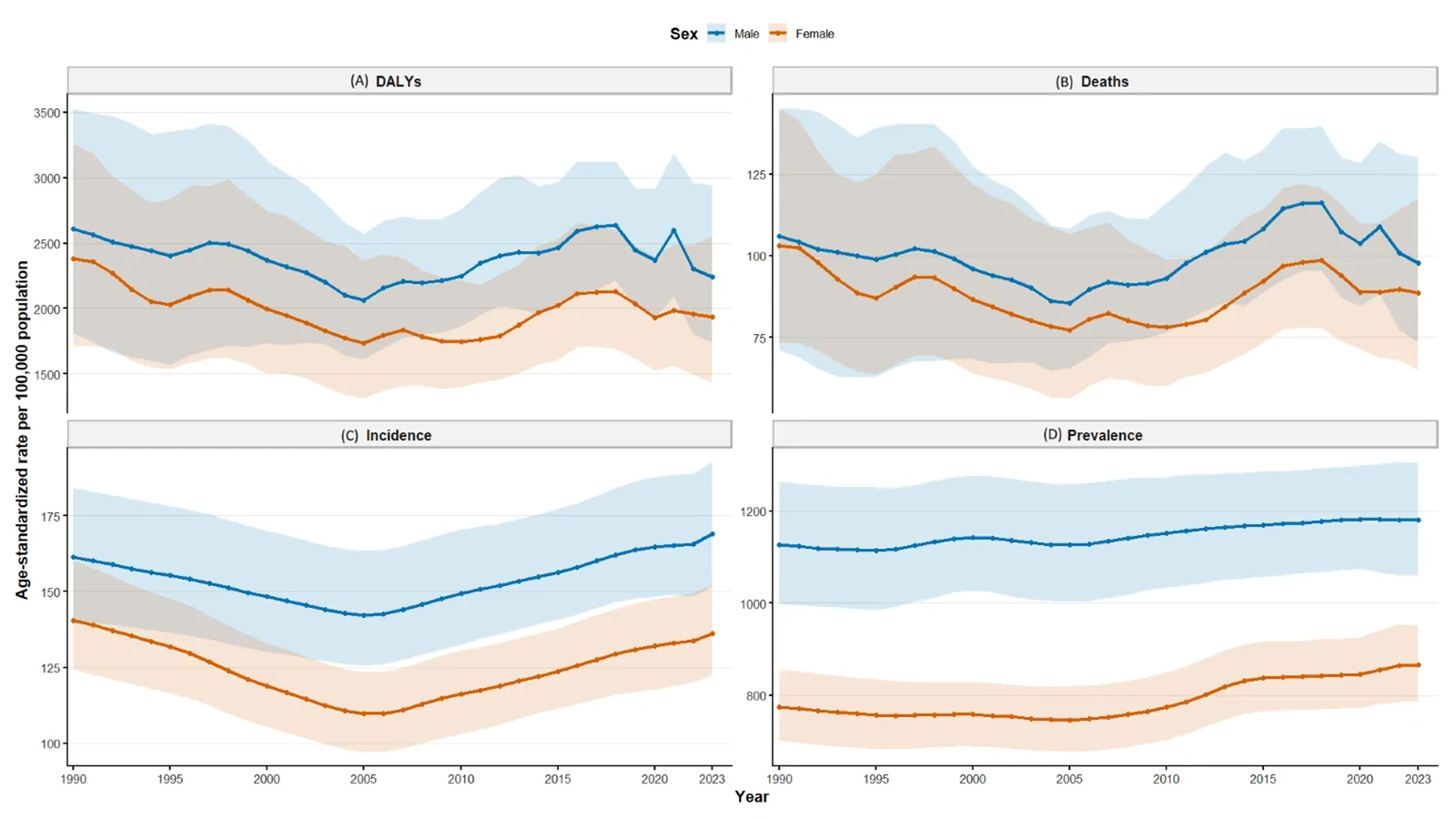
National trends in age-standardized (A) DALYs, (B) Deaths, (C) Incidence and (D) Prevalence rates for stroke burden among males and females in India, 1990-2023. Age-standardized rates are expressed per 100,000 population. Estimates were obtained from the Global Burden of Disease (GBD) Study 2023 [14]. Solid lines represent point estimates and shaded bands indicate 95% uncertainty intervals. DALYs: disability adjusted life years

A clear age gradient was seen for all the stroke outcomes in 2023, with incidence, prevalence, death, and DALY rates all increasing as people got older, the greatest increases occurring during middle adulthood and the highest rates being found in the oldest age groups. This trend was observed in both males and females, although men generally had higher rates of incidence, death, and DALYs in the adult and older age groups (Figure 3).

**Figure 3:**
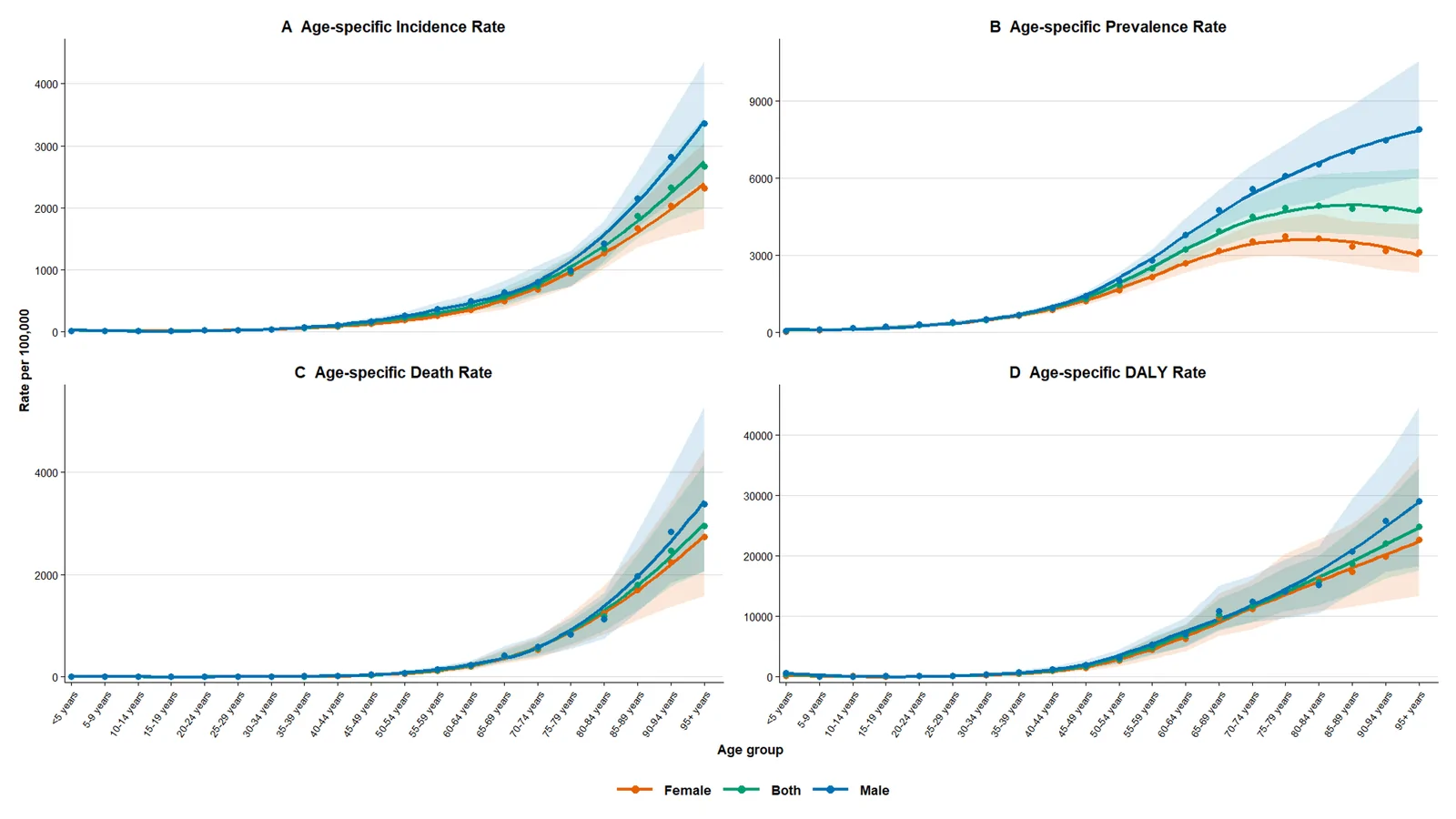
Age-specific (A) Incidence, (B) Prevalence, (C) Death and (D) DALY rates for stroke burden in India by sex, 2023. Age-specific rates are expressed per 100,000 population. Estimates were obtained from the Global Burden of Disease (GBD) Study 2023 [14]. Solid lines represent point estimates, and shaded bands indicate 95% uncertainty intervals. DALY: disability adjusted life year

Geographic distribution and temporal heterogeneity

In 2023, there was marked geographic variation in the burden of stroke among the states and UTs of India (see Figure 4). There were substantial differences in the distribution of ASRs of incidence, prevalence, death and DALYs between the states. Interestingly, the states which had the highest incidence were not always those that had the highest death or DALY rates.

**Figure 4:**
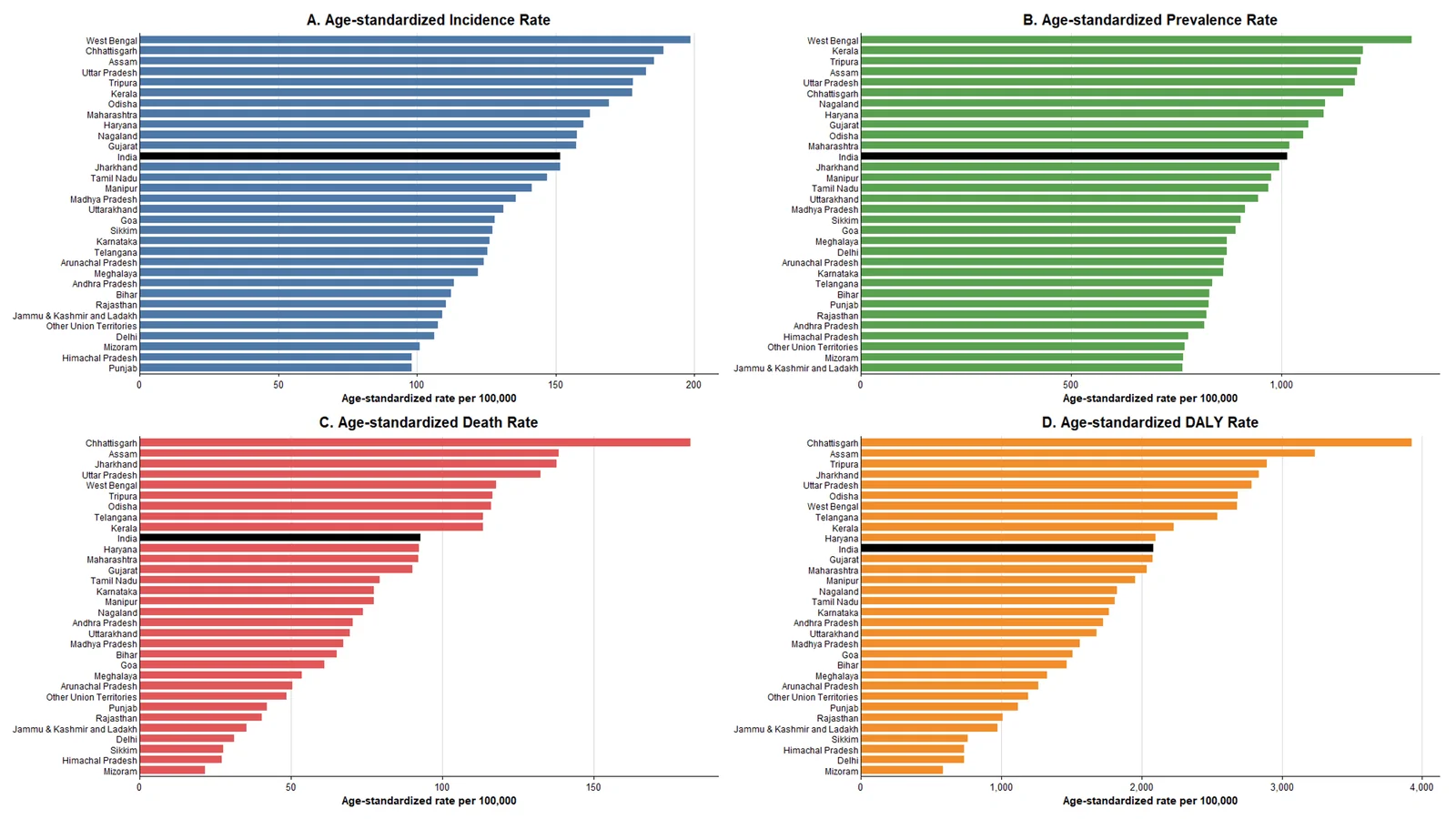
State-wise ranking of age-standardized (A) Incidence rate, (B) Prevalence rate, (C) Death rate and (D) DALY rate (with India as reference) for stroke burden in 2023. Estimates are presented for the administrative units defined by the Global Burden of Disease (GBD) Study 2023 [14], which may differ slightly from the current official administrative classification of India. In the GBD database, some recently created states or union territories are combined with their parent states or grouped as "Other Union Territories", and Jammu & Kashmir and Ladakh are reported together as a single unit. Consequently, the number and nomenclature of states and union territories shown in this figure may not exactly correspond to the present administrative divisions of India. Age-standardized rates are expressed per 100,000 population. DALY: disability adjusted life year

The extent and direction of temporal change also showed considerable variation from state to state (Figures 5, 6). The EAPCs at the state level exhibited different trends for the four outcomes, especially in the case of changes in death and DALYs. Hence, the national trend did not fully reflect the large variation in the temporal development of the stroke burden among different geographical areas. The location-specific EAPC estimates with corresponding 95% CIs for incidence, prevalence, DALYs, and deaths are presented in the Appendices (Appendix D), providing the numerical estimates underlying the observed geographical variation.

**Figure 5:**
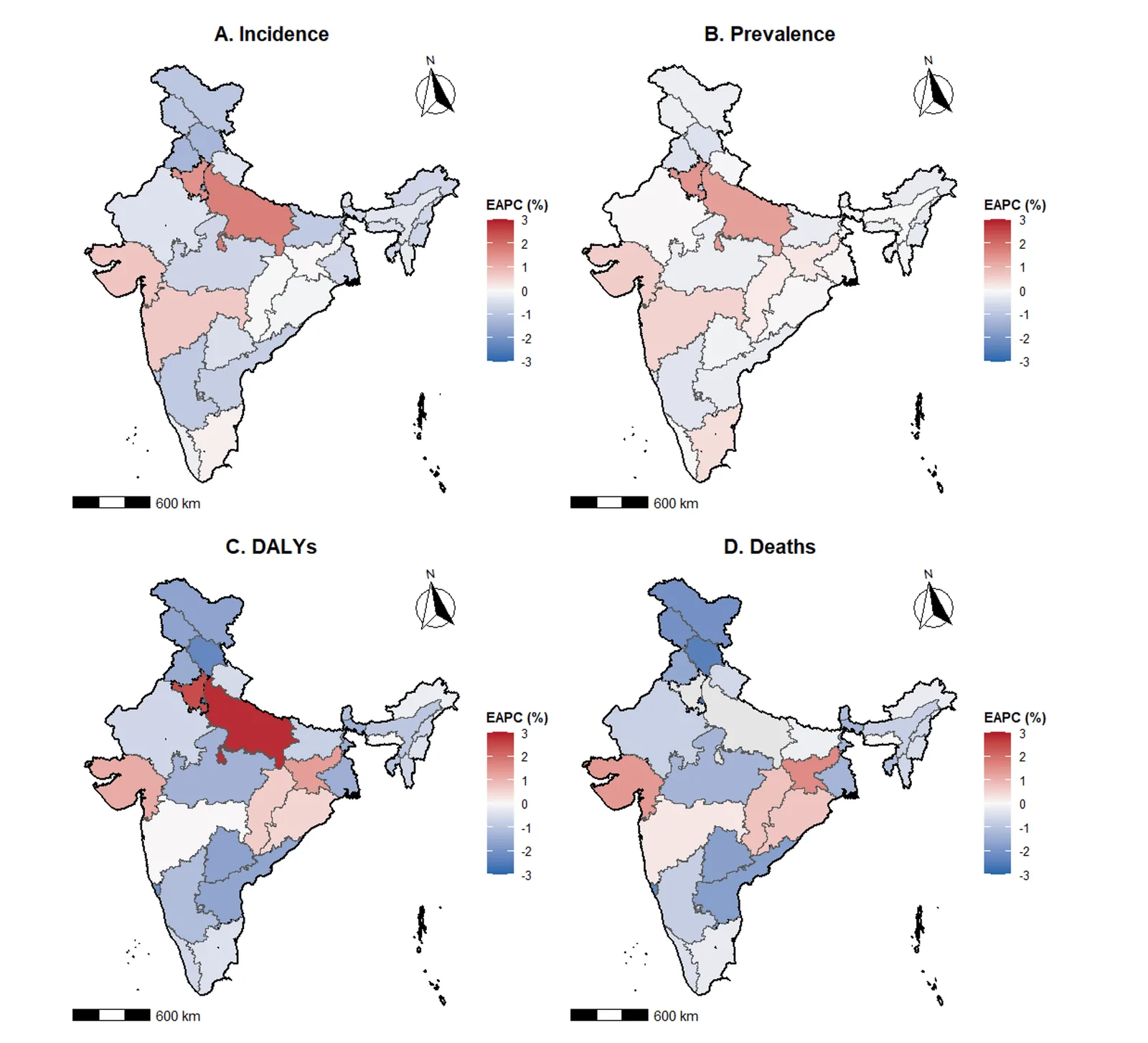
Geographic distribution of EAPC in age-standardized (A) Incidence, (B) Prevalence, (C) DALYs (D) Death rates for stroke across Indian states and union territories from 1990–2023. Maps show estimated annual percentage changes in age-standardized rates. Positive EAPCs indicate increasing trends, whereas negative EAPCs indicate declining trends. Panels A–D represent incidence, prevalence, DALYs, and deaths, respectively. Estimates were obtained from the Global Burden of Disease (GBD) Study 2023 [14]. EAPC: estimated annual percentage change; DALYs: disability adjusted life years Image Credit: The maps were created and visualized by the authors using R statistical software (R Foundation for Statistical Computing, Vienna, Austria) using an India shapefile obtained from the Survey of India (https://onlinemaps.surveyofindia.gov.in/Digital_Product_Show.aspx). The shapefile is freely available for use and can be accessed as an open/publicly available geospatial resource.

State-level EAPC analysis demonstrated considerable heterogeneity in the direction and magnitude of temporal changes in age-standardized incidence, prevalence, death, and DALY rates (Figure 6). Several states showed increasing trends, whereas others demonstrated declining or statistically stable trajectories.

**Figure 6:**
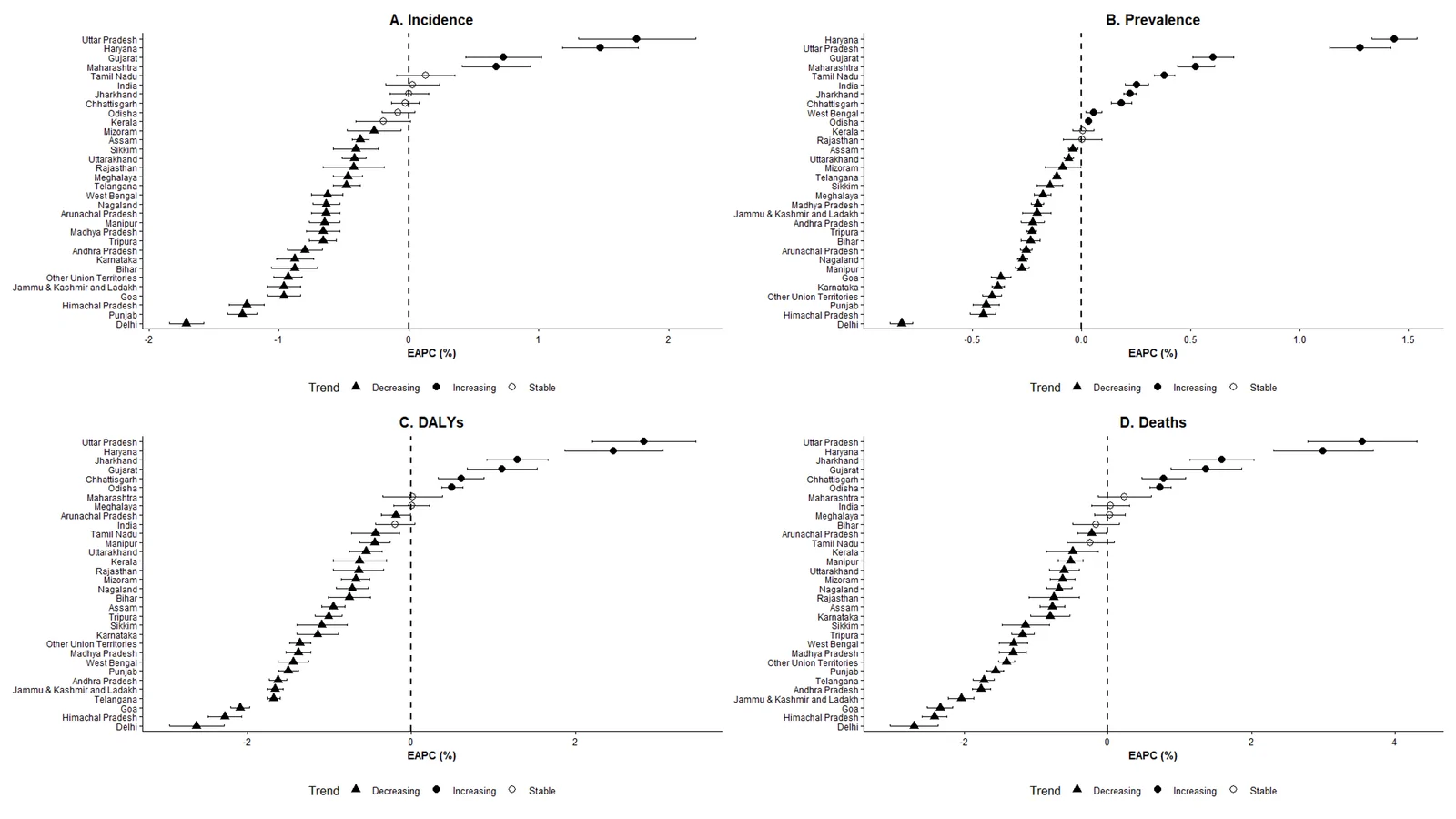
EAPC trend in age-standardized (A) Incidence, (B) Prevalence, (C) DALYs (D) Death rates by GBD-defined Indian locations from 1990 to 2023. Estimates are presented for the administrative units defined by the GBD Study 2023 [14], which may differ slightly from the current official administrative classification of India. In the GBD database, some recently created states or union territories are combined with their parent states or grouped as "Other Union Territories", and Jammu & Kashmir and Ladakh are reported together as a single unit. Consequently, the number and nomenclature of states and union territories shown in this figure may not exactly correspond to the present administrative divisions of India. EAPC: estimated annual percentage change; DALYs: disability adjusted life years; GBD: Global Burden of Disease

Joinpoint regression analysis

The analysis using Joinpoint regression revealed different national trends with regard to the major stroke outcomes (Figure 7). For the age-standardized incidence rate, there was no significant overall change from 1990 to 2023 (AAPC, −0.0195%; 95% CI −0.0405 to 0.0030; P=0.0796). On the other hand, both the age-standardized death rate and the DALY rate decreased significantly, the AAPC being −0.40% (95% CI −0.52 to −0.27; P<0.001) and −0.54% (95% CI −0.69 to −0.39; P<0.001), respectively. The decline in DALY rates was particularly steep during 2017-2023 (APC, −2.05%; 95% CI −3.02 to −1.34; P<0.001), compared with the overall AAPC of −0.54% (95% CI −0.69 to −0.39; P<0.001) for 1990-2023.

**Figure 7:**
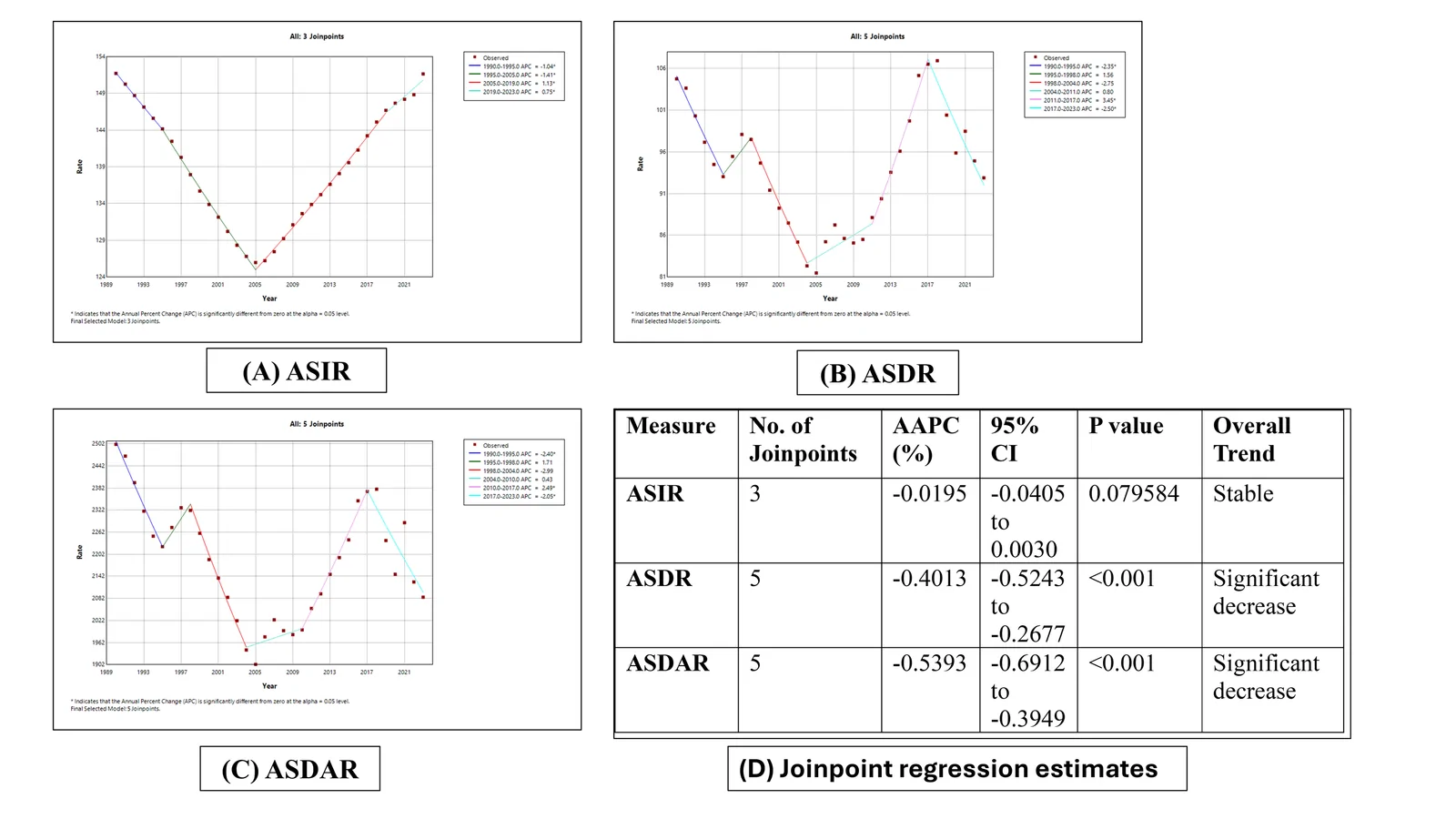
Temporal trends in age-standardized (A) incidence rate (ASIR), (B) death rate (ASDR) and (C) DALY rate (ASDAR) (D) Joinpoint regression analysis of stroke estimates in India from 1990 to 2023. Age-standardized rates are expressed per 100,000 population. Estimates were obtained from the Global Burden of Disease (GBD) Study 2023 [14]. Colored lines represent distinct temporal segments identified by the Joinpoint model. APCs are shown for each segment, while AAPCs summarize the overall trend. AAPC: average annual percent change; ASDAR: age-standardized disability adjusted life year rate; ASDR: age-standardized death rate; ASIR: age-standardized incidence rate; APC: annual percentage change

Stroke subtype patterns

Differences were found in the contributions of the major stroke subtypes to the total burden (Figure 8). The three subtypes showed distinct temporal patterns across the different outcomes, with ischemic stroke contributing most to incidence and prevalence, whereas the hemorrhagic subtypes contributed disproportionately to deaths and DALYs.

**Figure 8:**
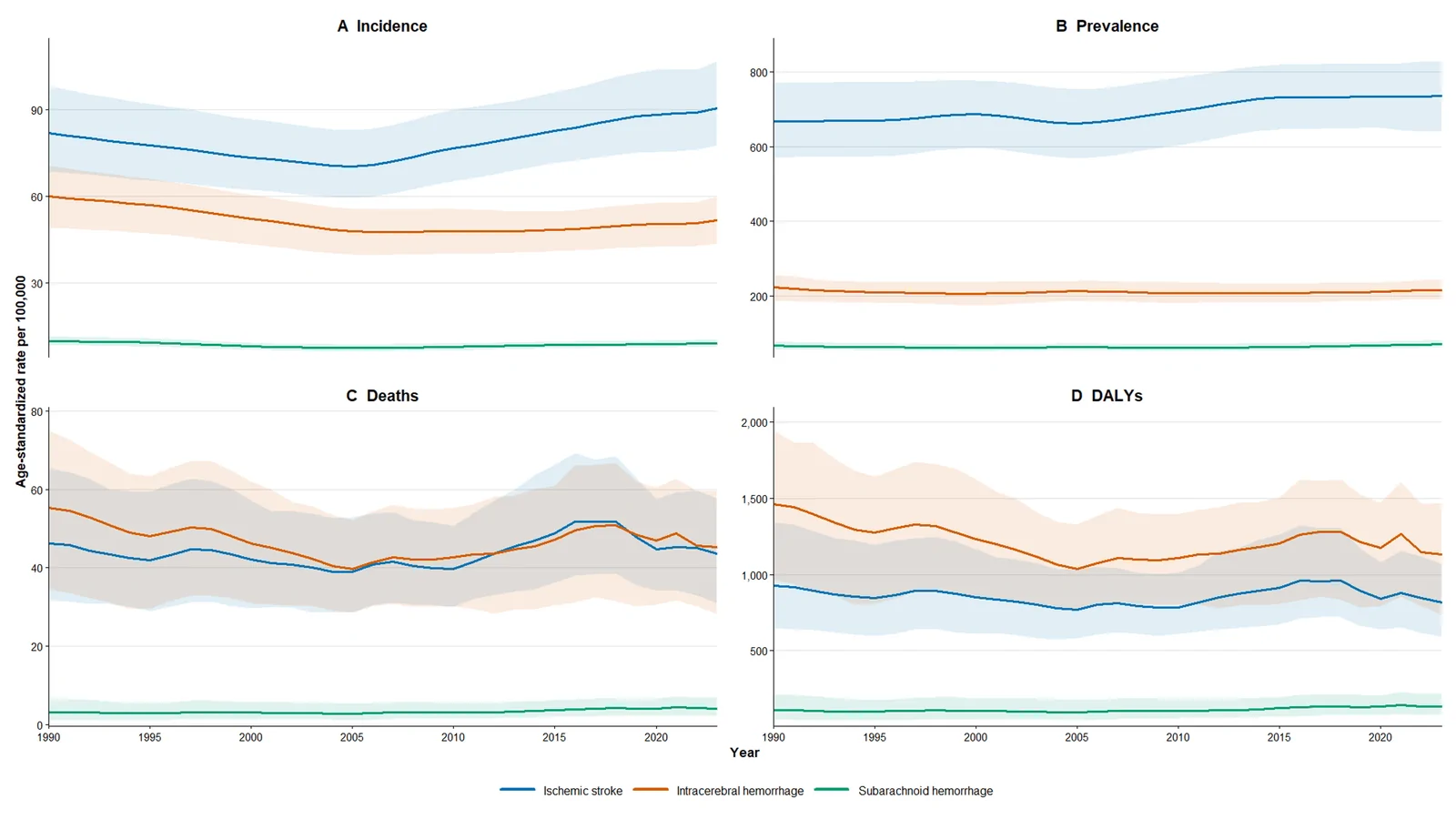
Temporal trends in age-standardized (A) Incidence, (B) Prevalence, (C) Death (D) DALY rates of stroke subtypes in India, 1990–2023. Age-standardized rates are expressed per 100,000 population. Estimates were obtained from the Global Burden of Disease (GBD) Study 2023 [14]. DALYs: disability adjusted life years

Decomposition of changes in absolute stroke burden

The Shapley decomposition showed that the increase in absolute stroke burden between 1990 and 2023 was mainly due to population growth and population ageing, although epidemiological change had a negative effect on all three outcomes (Table 2). With regard to incidence, population growth was responsible for 56.86% and population ageing for 45.45% of the increase observed, while epidemiological change offset 2.31% of the increase (Table 2). In the case of deaths, population growth and population ageing accounted for 59.21% and 61.67%, respectively, and epidemiological change contributed -20.88%. The demographic contribution was highest for DALYs, with population growth explaining 72.74% and population ageing 57.90%, while epidemiological change contributed -30.64% (Table 2).

**Table 2:** Shapley decomposition of demographic and epidemiological contributions to changes in absolute stroke burden. Estimates were obtained from the Global Burden of Disease (GBD) Study 2023 [14]. DALYs: disability adjusted life years

Outcome	Component	Absolute change	Contribution (%)
Incidence	Population growth	6,45,741	56.86
	Population ageing	5,16,252	45.45
	Epidemiological change	-26,242	-2.31
	Total	11,35,751	100.00
Deaths	Population growth	3,65,889	59.21
	Population ageing	3,81,064	61.67
	Epidemiological change	-1,29,020	-20.88
	Total	6,17,933	100.00
DALYs	Population growth	98,74,783	72.74
	Population ageing	78,60,936	57.90
	Epidemiological change	-41,60,051	-30.64
	Total	1,35,75,668	100.00

## Discussion

This study gives a recent assessment of the temporal, demographic, geographic, and subtype-specific stroke burden in India using GBD 2023 estimates from 1990 to 2023. Four findings are particularly important. First, the absolute stroke burden increased, with incidence, prevalence, deaths, and DALYs all increasing by more than 100% over the time period. In contrast, age-standardized incidence remained unchanged, while ASRs for death and DALYs declined. Second, stroke burden remained strongly age-dependent and consistently higher among males across most outcomes. Third, substantial heterogeneity was observed between states, with the geographical distribution of incidence differing from that of death and DALYs. Finally, the Shapley decomposition indicated that growth and ageing of the population were the principal contributors to the increase in stroke burden, whereas epidemiological change exerted a countervailing effect. The key message is therefore not that stroke risk has uniformly worsened in India, but that demographic expansion has translated relatively stable or declining age-specific risk into a larger absolute burden. This distinction is critical for interpreting the current stroke burden and for planning health services in a country undergoing fast demographic and epidemiological transition. The divergence between absolute numbers and ASRs was one of the most prominent findings of this analysis. Between 1990 and 2023, incident stroke cases increased by 155.1%, deaths by 148.2%, and DALYs by 109.4%, whereas age-standardized incidence changed by only −0.1%, mortality declined by 11.3%, and DALYs by 16.6%. This pattern is broadly consistent with previous GBD analyses from India, although magnitudes differ across GBD rounds and stroke definitions.

The combination of stable age-standardized incidence, declining age-standardized mortality, and increasing age-standardized prevalence is compatible with a growing pool of people living with stroke, potentially reflecting changes in survival and disease duration. However, because survival, case fatality, and disease duration were not directly analysed, these mechanisms remain hypotheses rather than demonstrated explanations.

A study using GBD 2019 data similarly demonstrated an increase in the absolute burden of ischemic stroke despite declines in several ASRs, including incidence, DALYs, and YLLs [3]. Likewise, earlier state-level analyses showed a marked increase in prevalent stroke burden over time, accompanied by marked differences between Indian states [5]. The consistency of similar findings across analyses suggests that the apparent paradox of rising stroke burden alongside improving standardized outcomes is a persistent feature of India's epidemiological transition rather than an artefact of a single GBD iteration.

Our findings extend this evidence through 2023 and provide a particularly clear demonstration of the demographic component of this divergence. The Shapley decomposition showed that population growth accounted for 56.86% of the increase in incident cases and 59.21% of the increase in deaths, while population ageing contributed a further 45.45% and 61.67%, respectively. For DALYs, the demographic contribution was even larger, with population growth and ageing accounting for 72.74% and 57.9% of the increase, respectively, while epidemiological change contributed −30.6%.

These findings are consistent with the larger demographic evidence from GBD studies showing that population expansion and ageing can increase the number of deaths and DALYs even when ASRs decline [18]. Importantly, the negative epidemiological component in our analysis should not be interpreted as a reduction in the importance of stroke. Rather, it indicates that changes in age-specific rates partially offset the burden generated by demographic expansion. Thus, India is experiencing epidemiologically favourable changes in ASRs that partially offset the increase in absolute burden generated by demographic expansion.

The age-standardized incidence rate was virtually unchanged between 1990 and 2023, and joinpoint regression similarly found no statistically significant overall trend (AAPC −0.0195%; 95% CI −0.0405 to 0.0030). This finding differs somewhat from the GBD 2019 analysis, which reported a significant decline in age-standardized stroke incidence in India, with an AAPC of −0.32% [7]. Such differences should not necessarily be interpreted as contradictory. GBD 2023 retrospectively re-estimated the historical series using updated data and modelling methods, meaning that estimates from different GBD rounds are not directly interchangeable [8].

More importantly, our annual trajectory suggests that incidence did not follow a simple linear pattern: age-standardized incidence declined during the earlier period but subsequently increased, resulting in little net change over the full time period. This pattern may indicate that earlier improvements in stroke prevention have been increasingly offset by persistent or emerging vascular risk exposures. Previous Indian analyses have documented an evolving risk environment characterized by persistent hypertension alongside increasing metabolic and dietary risks, while tobacco exposure has declined in several population groups [5,19]. These shifts provide a plausible epidemiological context for the later stabilization or increase in incidence, although the ecological nature of GBD data does not allow individual-level causal attribution. The contrast between stable incidence and declining mortality is compatible with improvements in stroke survival and case management, although the specific mechanisms cannot be established from these ecological data. Deaths and DALYs suggest improving outcomes, but residual disability remains substantial.

Age-standardized deaths declined by 11.3% and the age-standardized DALY rate by 16.6% over the time period, with Joinpoint regression confirming statistically significant long-term reductions in both deaths and DALYs. The AAPC was −0.40% for deaths and −0.54% for DALYs. Notably, the decline in DALY rates accelerated during the later period, with an annual decline of 2.05% during 2017-2023. The improvement in DALYs and deaths contrasts with the large increase in their absolute numbers and again highlights the importance of demographic structure. Previous GBD analyses have reported a similar distinction between age-specific improvements and increasing all-age burden [9]. Furthermore, deaths and DALYs capture different dimensions of stroke. DALYs incorporate both premature deaths and YLD; consequently, improved survival can reduce fatal outcomes while leaving a large population of stroke survivors requiring rehabilitation and long-term support [3,9]. This distinction has an important effect on health-system planning. A decline in age-standardized deaths should not be considered as evidence that the healthcare burden of stroke is diminishing. The increase in absolute DALYs, despite a decline in the age-standardized DALY rate, indicates a continuing and substantial population-level need for rehabilitation, secondary prevention, community-based disability management and long-term caregiver support.

The strong age gradient observed in 2023 is consistent with established evidence that stroke risk increases with advancing age. In our analysis, incidence, prevalence, deaths, and DALY rates increased progressively from adulthood and were highest in the older age groups, with the pattern evident in both sexes. Similar age-related increases have been demonstrated in India and internationally using GBD data [7,9,10]. The age gradient also helps explain the demographic decomposition results. As India's population increasingly shifts towards older age groups, even relatively modest improvements in age-specific stroke rates may be insufficient to prevent a rise in the number of stroke events. This reinforces the importance of interpreting age-standardized and absolute measures together rather than relying on either measure in isolation.

Stroke burden was also higher among men than women across the major outcomes. This is in agreement with the GBD 2021 analysis of stroke in India, which reported higher male incidence, deaths and DALY rates [9], as well as with the National Stroke Registry Programme, which reported higher age-standardized incidence among men than women [11]. However, previous GBD 2019 work found that the relative increase in ischemic stroke incidence and prevalence was greater among women than men as compared to deaths and DALYs, which were higher in men [3]. These observations are not necessarily inconsistent: higher male rates can coexist with faster proportional growth among women when baseline levels differ. Consequently, sex-specific prevention should not be limited to the group with the highest absolute rate.

One of the most policy-relevant findings was the pronounced geographical heterogeneity in stroke burden. States differed in age-standardized incidence, prevalence, deaths, and DALY rates, and states with higher incidence were not necessarily those with higher deaths or DALY rates. Temporal trends also differed considerably between states, with increasing, declining, and statistically stable trajectories occurring across different outcomes. This finding strongly corroborates previous evidence from India. The India State-Level Disease Initiative reported a 5.5-fold difference in crude stroke DALY rates between states in 2019 [4], while the earlier GBD cardiovascular analysis identified approximately six-fold variation in state-level stroke DALY rates [5]. The persistence of such heterogeneity across successive GBD rounds suggests that the national average conceals important subnational differences.

The dissociation between incidence and deaths is particularly informative. Differences in mortality may reflect not only differences in exposure to vascular risk factors but also variation in early recognition, access to emergency stroke care, treatment availability, secondary prevention and rehabilitation. The National Stroke Registry Programme, which covered five geographically distinct Indian areas, similarly demonstrated variation in stroke incidence and outcomes across rural and urban settings [11]. Therefore, geographic differences should not be interpreted as differences in biological risk. They may also reflect differences in healthcare access and ascertainment. This has implications for policy. A uniform national intervention is unlikely to produce equivalent benefits across all states. State-specific strategies integrating hypertension control, tobacco and dietary interventions, air-pollution reduction, timely stroke recognition, organized acute stroke care and rehabilitation are likely to be more appropriate than a single national approach.

The subtype analysis showed that ischemic stroke constituted the largest part of incidence and prevalence, whereas hemorrhagic stroke contributed disproportionately to deaths and DALYs. This pattern is consistent with findings from the National Stroke Registry Programme, in which ischemic stroke was the most common subtype across the participating registries [11]. The distinction is clinically and epidemiologically important. A predominance of ischemic stroke in incident and prevalent cases implies an increasing need for prevention and management of atherosclerotic and cardioembolic risk factors. Conversely, the disproportionate contribution of hemorrhagic stroke to death and disability highlights the continuing importance of aggressive blood-pressure control. Previous GBD analyses have consistently identified high systolic blood pressure as one of the major modifiable contributors to stroke burden in India [4,5,19]. Thus, the subtype findings enhance the importance of hypertension prevention not only for reducing the overall number of strokes but also for addressing the forms of stroke associated with particularly severe outcomes.

Taken together, our findings point towards a shift in the strategic focus of stroke control in India. The country cannot rely solely on reducing ASRs; the absolute number of people affected will remain large because of population growth and ageing. At the same time, the stability of incidence and decline in deaths and DALYs indicate that further gains are possible. Prevention should therefore prioritize high-impact modifiable risk factors, particularly hypertension, while addressing dietary risk, metabolic abnormalities, tobacco exposure, obesity, and air pollution. High systolic blood pressure has repeatedly emerged as the leading attributable risk for stroke in India [4,19]. However, the geographical variation in risk profiles suggests that interventions should be adapted to local epidemiological conditions rather than implemented as identical packages across all states. The health system must also prepare for a growing population of stroke survivors. The increase in absolute DALYs despite a decline in the age-standardized DALY rate indicates that improvements in survival do not eliminate the need for long-term care. Expanding stroke units and emergency pathways should therefore occur alongside rehabilitation services, secondary prevention and community-based follow-up.

Strengths

This study has some major strengths. It uses the latest GBD 2023 estimates and examines the complete 1990-2023 period, allowing comparison of incidence, prevalence, deaths and DALYs within a common analytical framework. The combination of age- and sex-specific analyses, geographical region-level assessment, Joinpoint regression, subtype analysis and Shapley decomposition provides complementary perspectives on both the magnitude and structure of the changing burden.

Limitations

Several limitations should also be considered. First, GBD estimates are model-based and are subject to uncertainty arising from data availability, cause-of-death classification, diagnostic and ascertainment practices, and modelling assumptions; the completeness and quality of underlying data may also have varied over time. Second, comparisons with earlier GBD studies should be interpreted cautiously because successive GBD rounds incorporate updated data and methods and retrospectively revise historical estimates [8]. Third, state-level estimates may conceal substantial within-location heterogeneity and differences in healthcare access, while some location-specific estimates may be less stable, particularly for smaller populations or less frequently measured outcomes. Fourth, the SEs used for Joinpoint regression were approximated from GBD UIs rather than derived from conventional observed-data SEs; therefore, the resulting trend estimates should be interpreted cautiously, and serial correlation between annual estimates could not be fully accounted for. Fifth, the Shapley decomposition identifies demographic and epidemiological contributions to changes in absolute burden but does not establish causal mechanisms. Finally, as an ecological analysis, the study cannot infer individual-level associations or attribute observed temporal changes to specific risk factors, lifestyle factors, health policies, or interventions; uncertainty in subtype-specific GBD estimates may also affect the precision of subtype-specific comparisons.

## Conclusions

The stroke burden in India has evolved in the context of substantial demographic and epidemiological transition, with population growth and ageing emerging as important contributors of the increasing absolute burden. At the same time, age-standardized incidence remained essentially stable, while age-standardized mortality and DALY rates declined, indicating declines in these measures of stroke burden. The persistence of pronounced age- and sex-related differences, substantial geographical heterogeneity, and the disproportionate contribution of hemorrhagic stroke to mortality and disability underscores the need for a more targeted and context-specific stroke-control strategy. National efforts should therefore be complemented by geographically and demographically tailored interventions, with particular emphasis on prevention and control of modifiable risk factors, especially hypertension.

Health-system planning must simultaneously account for the continuing absolute burden of stroke. Strengthening primary prevention, timely access to acute stroke care, secondary prevention, rehabilitation, and long-term community-based support will be essential. Overall, the findings highlight that declining standardized rates alone should not be interpreted as a reduction in the public health challenge posed by stroke; demographic change must remain central to future stroke prevention, service planning, and resource allocation in India.

## Appendices

Appendix A 

**Table 3:** GBD 2023 data selection criteria for national, sex-specific, age-specific, and stroke subtype analyses, India, 1990–2023 Reference: [14] DALYs: disability adjusted life years; GBD: Global Burden of Disease

Parameter	Selection for National trend analysis	Selection for Sex specific analysis	Selection for Age specific analysis	Selection for Sub-type specific analysis
GBD estimate	Cause of death or injury	Cause of death or injury	Cause of death or injury	Cause of death or injury
Measures	Incidence, Prevalence, Deaths, DALYs	Incidence, Prevalence, Deaths, DALYs	Incidence, Prevalence, Deaths, DALYs	Incidence, Prevalence, Deaths, DALYs
Metrics	Number + Rate	Rate	Rate	Rate
Cause	Stroke	Stroke	Stroke	Ischemic stroke, Intracerebral hemorrhage, and Subarachnoid hemorrhage
Locations	India	India	India	India
Age	All ages, Age-standardized	Age-standardized	<5, 5–9, 10–14, 15–19, 20–24, 25–29, 30–34, 35–39, 40–44, 45–49, 50–54, 55–59, 60–64, 65–69, 70–74, 75–79, 80–84, 85–89, 90–94, and ≥95 years	Age-standardized
Sex	Both	Male Female Both	Male Female Both	Both
Years	1990–2023	1990–2023	1990–2023	1990–2023

Appendix B 

**Table 4:** Population data selection for Shapley decomposition analysis of stroke burden, India, 1990–2023 GBD: Global Burden of Disease

Parameter	Selection for Shapley decomposition
GBD estimate	Population
Measures	Population
Metrics	Number
Locations	India
Age	<5, 5–9, 10–14, 15–19, 20–24, 25–29, 30–34, 35–39, 40–44, 45–49, 50–54, 55–59, 60–64, 65–69, 70–74, 75–79, 80–84, 85–89, 90–94, and ≥95 years
Sex	Both
Years	1990–2023

Appendix C 

**Table 5:** Joinpoint regression model specifications for temporal trend analysis of stroke burden, India, 1990–2023

Parameter	Value
Software	Joinpoint Regression Program, version 6.1.0 (National Cancer Institute, Bethesda, Maryland, United States)
Maximum joinpoints	6
Minimum joinpoints	0
Minimum observations between two joinpoints	2
Transformation	Log-transformed {ln(y) = xb}
Model selection	Permutation test
Statistical testing	Significance level: 0.0500

Appendix D 

**Table 6:** Estimated annual percentage change (EAPC) in age-standardized rates by state, 1990–2023. Estimates are presented for the administrative units defined by the Global Burden of Disease (GBD) Study 2023 [14], which may differ slightly from the current official administrative classification of India. In the GBD database, some recently created states or union territories are combined with their parent states or grouped as "Other Union Territories", and Jammu & Kashmir and Ladakh are reported together as a single unit. Consequently, the number and nomenclature of states and union territories shown in this figure may not exactly correspond to the present administrative divisions of India. Age-standardized rates are expressed per 100,000 population. DALYs: disability adjusted life years

State	Incidence, EAPC (95%CI)	Prevalence, EAPC (95%CI)	DALYs, EAPC (95%CI)	Deaths, EAPC (95%CI)
Andhra Pradesh	-0.80 (-0.93 to -0.67)	-0.22 (-0.28 to -0.17)	-1.63 (-1.73 to -1.52)	-1.76 (-1.89 to -1.63)
Arunachal Pradesh	-0.64 (-0.75 to -0.53)	-0.25 (-0.28 to -0.23)	-0.19 (-0.37 to -0.01)	-0.22 (-0.42 to -0.03)
Assam	-0.37 (-0.44 to -0.31)	-0.04 (-0.06 to -0.02)	-0.95 (-1.09 to -0.81)	-0.77 (-0.94 to -0.60)
Bihar	-0.88 (-1.06 to -0.70)	-0.24 (-0.28 to -0.19)	-0.76 (-1.02 to -0.50)	-0.17 (-0.49 to 0.16)
Chhattisgarh	-0.02 (-0.13 to 0.08)	0.18 (0.14 to 0.23)	0.61 (0.33 to 0.88)	0.78 (0.47 to 1.08)
Delhi	-1.71 (-1.85 to -1.58)	-0.83 (-0.88 to -0.77)	-2.62 (-2.95 to -2.28)	-2.70 (-3.03 to -2.37)
Goa	-0.96 (-1.09 to -0.83)	-0.37 (-0.42 to -0.32)	-2.09 (-2.20 to -1.97)	-2.34 (-2.51 to -2.16)
Gujarat	0.73 (0.44 to 1.02)	0.60 (0.51 to 0.70)	1.11 (0.68 to 1.53)	1.37 (0.88 to 1.86)
Haryana	1.47 (1.18 to 1.77)	1.43 (1.33 to 1.54)	2.46 (1.87 to 3.06)	3.00 (2.31 to 3.70)
Himachal Pradesh	-1.25 (-1.38 to -1.11)	-0.45 (-0.51 to -0.39)	-2.27 (-2.48 to -2.07)	-2.41 (-2.59 to -2.24)
India	0.03 (-0.18 to 0.24)	0.25 (0.20 to 0.31)	-0.20 (-0.44 to 0.04)	0.03 (-0.23 to 0.30)
Jammu & Kashmir and Ladakh	-0.96 (-1.09 to -0.83)	-0.21 (-0.27 to -0.14)	-1.66 (-1.76 to -1.56)	-2.04 (-2.22 to -1.87)
Jharkhand	0.00 (-0.14 to 0.15)	0.22 (0.19 to 0.25)	1.29 (0.92 to 1.67)	1.59 (1.14 to 2.03)
Karnataka	-0.88 (-1.02 to -0.73)	-0.38 (-0.41 to -0.36)	-1.14 (-1.39 to -0.89)	-0.80 (-1.07 to -0.53)
Kerala	-0.20 (-0.41 to 0.01)	0.01 (-0.04 to 0.06)	-0.63 (-0.95 to -0.30)	-0.49 (-0.85 to -0.13)
Madhya Pradesh	-0.66 (-0.79 to -0.53)	-0.20 (-0.23 to -0.17)	-1.37 (-1.53 to -1.22)	-1.32 (-1.51 to -1.14)
Maharashtra	0.67 (0.41 to 0.94)	0.52 (0.44 to 0.61)	0.02 (-0.35 to 0.38)	0.24 (-0.13 to 0.60)
Manipur	-0.65 (-0.77 to -0.53)	-0.27 (-0.31 to -0.24)	-0.45 (-0.63 to -0.26)	-0.52 (-0.69 to -0.34)
Meghalaya	-0.47 (-0.58 to -0.36)	-0.18 (-0.22 to -0.14)	0.00 (-0.21 to 0.22)	0.03 (-0.19 to 0.24)
Mizoram	-0.27 (-0.48 to -0.06)	-0.09 (-0.17 to -0.00)	-0.68 (-0.85 to -0.50)	-0.63 (-0.80 to -0.46)
Nagaland	-0.63 (-0.74 to -0.53)	-0.27 (-0.29 to -0.25)	-0.72 (-0.92 to -0.52)	-0.68 (-0.85 to -0.50)
Odisha	-0.08 (-0.20 to 0.04)	0.03 (0.02 to 0.05)	0.50 (0.37 to 0.62)	0.73 (0.58 to 0.88)
Other Union Territories	-0.93 (-1.04 to -0.82)	-0.41 (-0.45 to -0.37)	-1.36 (-1.49 to -1.23)	-1.41 (-1.53 to -1.30)
Punjab	-1.28 (-1.40 to -1.17)	-0.44 (-0.50 to -0.38)	-1.50 (-1.62 to -1.38)	-1.57 (-1.68 to -1.45)
Rajasthan	-0.42 (-0.66 to -0.19)	0.00 (-0.08 to 0.09)	-0.64 (-0.95 to -0.34)	-0.75 (-1.10 to -0.40)
Sikkim	-0.41 (-0.58 to -0.23)	-0.15 (-0.21 to -0.09)	-1.09 (-1.39 to -0.78)	-1.14 (-1.47 to -0.82)
Tamil Nadu	0.13 (-0.09 to 0.36)	0.38 (0.34 to 0.43)	-0.44 (-0.73 to -0.14)	-0.24 (-0.57 to 0.09)
Telangana	-0.48 (-0.58 to -0.38)	-0.11 (-0.13 to -0.10)	-1.68 (-1.76 to -1.60)	-1.73 (-1.88 to -1.58)
Tripura	-0.66 (-0.77 to -0.56)	-0.23 (-0.25 to -0.21)	-1.01 (-1.17 to -0.84)	-1.18 (-1.34 to -1.02)
Uttar Pradesh	1.76 (1.31 to 2.21)	1.28 (1.14 to 1.42)	2.84 (2.21 to 3.47)	3.54 (2.78 to 4.30)
Uttarakhand	-0.42 (-0.51 to -0.33)	-0.06 (-0.08 to -0.04)	-0.55 (-0.75 to -0.35)	-0.61 (-0.81 to -0.40)
West Bengal	-0.63 (-0.75 to -0.51)	0.06 (0.02 to 0.09)	-1.44 (-1.62 to -1.25)	-1.31 (-1.51 to -1.12)

## References

[1] Tsao CW, Aday AW, Almarzooq ZI (2023). Heart disease and stroke statistics—2023 update: a report from the American Heart Association. Circulation.

[2] Rautalin I, Volovici V, Stark BA (2025). Global, regional, and national burden of nontraumatic subarachnoid hemorrhage: the Global Burden of Disease Study 2021. JAMA Neurol.

[3] Goyal AD, Gajjar AA, Muhammad N (2025). A three-decade analysis of ischemic stroke in India: mortality, morbidity, and risk factors using the Global Burden of Diseases study from 1990 to 2019. J Clin Med.

[4] (2021). The burden of neurological disorders across the states of India: the Global Burden of Disease Study 1990-2019. Lancet Glob Health.

[5] (2018). The changing patterns of cardiovascular diseases and their risk factors in the states of India: the Global Burden of Disease Study 1990-2016. Lancet Glob Health.

[6] (2024). Global incidence, prevalence, years lived with disability (YLDs), disability-adjusted life-years (DALYs), and healthy life expectancy (HALE) for 371 diseases and injuries in 204 countries and territories and 811 subnational locations, 1990-2021: a systematic analysis for the Global Burden of Disease Study 2021. Lancet.

[7] Ji X, Tao M, Guo H-Y (2023). Age-period-cohort analysis of stroke incidence in China and India from 1990 to 2019 and predictions up to 2042 [PREPRINT]. Research Square.

[8] (2025). Global burden of 292 causes of death in 204 countries and territories and 660 subnational locations, 1990-2023: a systematic analysis for the Global Burden of Disease Study 2023. Lancet.

[9] Behera DK, Rahut DB, Mishra S (2024). Analyzing stroke burden and risk factors in India using data from the Global Burden of Disease Study. Sci Rep.

[10] Wang Z, Liu Y, Qie R, Hu Y (2025). Comparative analysis of stroke burden between ages 20-54 and over 55 years: based on the Global Burden of Disease study 2019. BMC Public Health.

[11] Rangamani S, Huliyappa D, Kulothungan V (2024). Stroke incidence, mortality, subtypes in rural and urban populations in five geographic areas of India (2018-2019): results from the National Stroke Registry Programme. Lancet Reg Health Southeast Asia.

[12] Song D, Cai R, Du Y (2026). Age-period-cohort analysis of stroke and subtype burdens from 1990 to 2021. Nutr Metab Cardiovasc Dis.

[13] Stevens GA, Alkema L, Black RE (2016). Guidelines for accurate and transparent health estimates reporting: the GATHER statement. Lancet.

[14] Global Burden of Disease Study 2023 (GBD (2026). Institute for Health Metrics and Evaluation/Global Health Data Exchange (GHDx): Global Burden of Disease (GBD) results, 2023. https://vizhub.healthdata.org/gbd-results/.

[15] Kaushik R, Vashistha G (2026). Injury burden in India, 1990 to 2023: national and subnational trends from the global burden of disease 2023 study. Cureus.

[16] Zhu J, Li S, Li X, Wang L, Du L, Qiu Y (2024). Impact of population ageing on cancer-related disability-adjusted life years: a global decomposition analysis. J Glob Health.

[17] Shorrocks AF (2013). Decomposition procedures for distributional analysis: a unified framework based on the shapley value. J Econ Inequal.

[18] (2025). Global age-sex-specific all-cause mortality and life expectancy estimates for 204 countries and territories and 660 subnational locations, 1950-2023: a demographic analysis for the Global Burden of Disease Study 2023. Lancet.

[19] Nath M, Tangri P, Arora B (2026). Evolving epidemiology of stroke in India: burden, inequalities, and risk factors from 1990 to 2023 with projections to 2035 [PREPRINT]. medRxiv.

